# Short-term memory capacity analysis of Lu_3_Fe_4_Co_0.5_Si_0.5_O_12_-based spin cluster glass towards reservoir computing

**DOI:** 10.1038/s41598-023-32084-8

**Published:** 2023-03-31

**Authors:** Zhiqiang Liao, Hiroyasu Yamahara, Kenyu Terao, Kaijie Ma, Munetoshi Seki, Hitoshi Tabata

**Affiliations:** 1 Department of Electrical Engineering and Information Systems, Graduate School of Engineering, The University of Tokyo, 7-3-1 Hongo, Bunkyo-Ku, Tokyo, 113-8656 Japan; 2 Department of Bioengineering, Graduate School of Engineering, The University of Tokyo, 7-3-1 Hongo, Bunkyo-Ku, Tokyo, 113-8656 Japan; 3 Center for Spintronics Research Network, Graduate School of Engineering, The University of Tokyo, 7-3-1 Hongo, Bunkyo-Ku, Tokyo, 113-8656 Japan

**Keywords:** Magnetic properties and materials, Nonlinear phenomena

## Abstract

Reservoir computing is a brain heuristic computing paradigm that can complete training at a high speed. The learning performance of a reservoir computing system relies on its nonlinearity and short-term memory ability. As physical implementation, spintronic reservoir computing has attracted considerable attention because of its low power consumption and small size. However, few studies have focused on developing the short-term memory ability of the material itself in spintronics reservoir computing. Among various magnetic materials, spin glass is known to exhibit slow magnetic relaxation that has the potential to offer the short-term memory capability. In this research, we have quantitatively investigated the short-term memory capability of spin cluster glass based on the prevalent benchmark. The results reveal that the magnetization relaxation of Co, Si-substituted Lu_3_Fe_5_O_12_ with spin glass behavior can provide higher short-term memory capacity than ferrimagnetic material without substitution. Therefore, materials with spin glass behavior can be considered as potential candidates for constructing next-generation spintronic reservoir computing with better performance.

## Introduction

With the society transitioning into the high-speed informatization era, artificial intelligence technology has become crucial as an efficient method of data processing. However, the separation of memory and computation of traditional computers restricts the embedded interaction between artificial intelligence systems and modern devices and significantly increases the power consumption of computing^1^. In recent years, to overcome this bottleneck, researchers have developed neuromorphic devices with high integration and low power consumption by simulating the function of neurons and synapses^2–5^. Among the existing neuromorphic devices, a framework called reservoir computing has been favored by researchers^6–8^. The basic principle is based on mapping data to a high-dimensional space using large reservoir units and learning its characteristics^9^. For hardware devices, a large number of reservoir units in the reservoir computing software can be easily simulated by time multiplexing a single unit^10^. In the training process, only the output layer data must be trained using simple linear regression^11^. In essence, a reservoir computing system is mainly required to have two characteristics: short-term memory ability and nonlinearity^12^. Short-term memory refers to a small amount of external input information that remains a transient active state in the memory system for a short time ^13^. The short-term memory ability can cause the historical information entering the reservoir to gradually decay and disappear with time. As another key characteristic, nonlinearity can be easily realized in various physical devices. Therefore, reservoir computing is simple in terms of training, fast convergence, and easy physical implementation.

So far, physical reservoir computing has been developed based on various systems^14–16^, including electronic systems^17,18^, mechanical systems^19,20^, optical systems^21,22^, spintronics devices^23,24^. Among them, magnetic-tunnel-junction based spintronic devices are considered to be a promising choice for physical reservoir computing, owing to their non-volatility, read/write endurance, high-speed operation, low power consumption and low size^25–28^. Besides, spin wave^29,30^, skyrmion^31^, antiferromagnetic systems^32^ are also used for constructing spintronics reservoir computing systems with different physical principles. Nontheless, whether it is based on magnetic tunnel junction or other spintronics systems, the present spintronic reservoir computing realizes the required short-term memory ability through the relaxation process originates from certain physical phenomena^27,28^ or the delay module in the system^29,30^. The nonlinearity can be simply provided by the magnetic materials and the spin dynamics^28–30^. Interestingly, when the short-term memory and nonlinearity are simultaneously provided from multiple components, the abilities of the entire system intensify more than those of a single component^28,29,33^. Therefore, in an ideal case, it is expected that all components in spintronic reservoir computing can provide short-term memory ability and nonlinearity. However, in the existing studies, the magnetic characteristics of ferromagnets used in spintronic devices mainly contribute to the nonlinearity^28,29^. Among various magnetic materials, spin glasses are disordered magnets, where spin-freezing state occurs below the spin-freezing temperature (*T*_g_), and exhibit characteristic memory and aging effects related to the slow magnetic relaxation. From the perspective of the mean-field theory, this characteristic property of spin glass is explained by the multi-valley potential, which is caused by the randomness of incoming atoms and the frustration between spin interactions^34,35^. Although a number of spin glasses have been reported to show memory effects, particularly pertaining to memorizing the thermal and/or magnetic field history^36–38^, there are no quantitative studies discussing their connection with short-term memory ability in physical reservoir computing.

To address this research gap, we quantitatively tested the short-term memory capacity of Lu_3_Fe_4_Co_0.5_Si_0.5_O_12_ (LFCS), which has demonstrated spin cluster glass behaviors of memory effect with high *T*_g_^39^, through the short-term memory task commonly used by reservoir computing. It is well-known that there is an upper limit on the amount of new information for the memory system to remember in a short time ^40^. This limit is the short-term memory capacity, also known as memory span. For a reservoir computing system, the memory span can be quantitatively evaluated by two different methods. The first is benchmarking the system on short-term memory task with quantitatively defined short-term memory capacity as an index^41^. The second method is using information processing capacity to comprehensively evaluate the nonlinear and linear capacities of the binary input-driven reservoir computing system^42,43^. Because we mainly focus on the short-term memory capacity of spin glass, the first method is chose in this work. Numerical simulation and experimental results showed that the short-term memory capacity of LFCS magnetization is higher than that of typical ferrimagenetic material of Lu_3_Fe_5_O_12_ (LuIG). Specifically, when the input binary sequence is mapped from {0, 1} to applied magnetic field {0, 100 Oe} and the working temperature is 150 K immediately below *T*_g_, the magnetization of spin glass has an short-term memory capacity around 2. In contrast, LuIG does not have an effective short-term memory capability. We also found that the short-term memory ability in the experimental results is attenuated compared with the ideal simulation that can be attributed to the relaxation introduced by noise and the measurement system. In addition, the relationship between the amplitude of external write magnetic field for LFCS and the short-term memory ability is explored. It was revealed that the short-term memory ability of LFCS is suitable for utilization under a field strength of approximately 100 Oe. Because the aging memory behaviors of LFCS are common in the spin glass family ^37,44,45^, the conclusions of this work can be similarly extended to typical spin glass materials. These results contribute a quantitative connection between the spin glass behavior and the short-term memory capacity of reservoir computing. Hence, this work is expected to offer a potentail material candidate for improving the spintronics reservoir computing.

## Material and methods

### Spin cluster glass of Lu_3_Fe_4_Co_0.5_Si_0.5_O_12_

The sample preparation and spin cluster glass behaviors of LFCS have already been reported^35,36,46^. The general chemical formula of rare-earth iron garnet is (*R*_3_) [Fe_2_] _*a*_[Fe_3_]_*d*_O_12_ (where *R* denotes the rare-earth elements). Octahedral *a*-sites and tetragonal *d*-sites are occupied by 16 and 24 Fe^3+^ ions, respectively, whereas the dodecahedral *c*-sites are occupied by 24 *R*^3+^ ions. Among various rare-earth iron garnets, magnetic moments in Fe^3+^ in octahedral and tetragonal sites are dominant in the magnetization of lutetium iron garnet (Lu_3_Fe_5_O_12_, LuIG) because the 4f. level is filled in Lu^3+^. The magnetic moments of Fe^3+^ in octahedral and tetragonal sites are antiferromagnetically aligned with each other; hence, the net moment corresponds to that for one Fe^3+^ per formula unit, resulting in ferrimagnetism. Among various magnetic materials, LuIG and YIG (Y_3_Fe_5_O_12_) show exceptionally low damping constants for spin wave propagation, which means long lifetime and free path. The small magnetic loss is necessary for spin wave application including reservoir computing. Although typical magnetic materials such as permalloy, CoFeB, and Heusler alloys show the damping constants of 10^–3^, LuIG and YIG show one digit or more smaller values as 10^–5^–10^–4^^47^. Both LuIG and YIG are garnet-structured magnetic insulator and composed of the spectroscopic S-state of all the ions that carry the magnetic moment, which result in very small spin–orbit interaction and magnetic loss. Therefore, LuIG and YIG are commonly used candidates for spin wave devices. Because spin glass behaviors have been reported in Co and Si-doped LuIG in addition to the characteristics of spin wave propagation ^39^, we selected LuIG in this work. According to previous studies^39^, Co^2+^ and Si^4+^ prefer to substitute the octahedral and tetrahedral sites, respectively. The incorporation of anisotropic Co^2+^ and nonmagnetic Si^4+^ induces randomness and frustration of magnetic interactions. Thus, similar spin cluster glass behavior as that previously reported in spinel-type ferrimagnets, was observed^48,49^. LuIG and LFCS films were deposited on Y_3_Al_5_O_12_ (111) substrates through pulsed laser deposition using an ArF excimer laser. The source targets were prepared using a conventional solid-state reaction. Powders of Lu_2_O_3_ (99.9%), *α*-Fe_2_O_3_ (99.99%), CoO (99.9%), and SiO_2_ (99.9%) were mixed stoichiometrically and sintered at 1400 °C for 6 h. During the film growth, the substrate temperature and ambient oxygen pressure were maintained at 750 °C and 1 Pa, respectively. The as-grown films were post-annealed in air at 800 °C for 2 h to improve their crystallinity.

**Figure 1:**
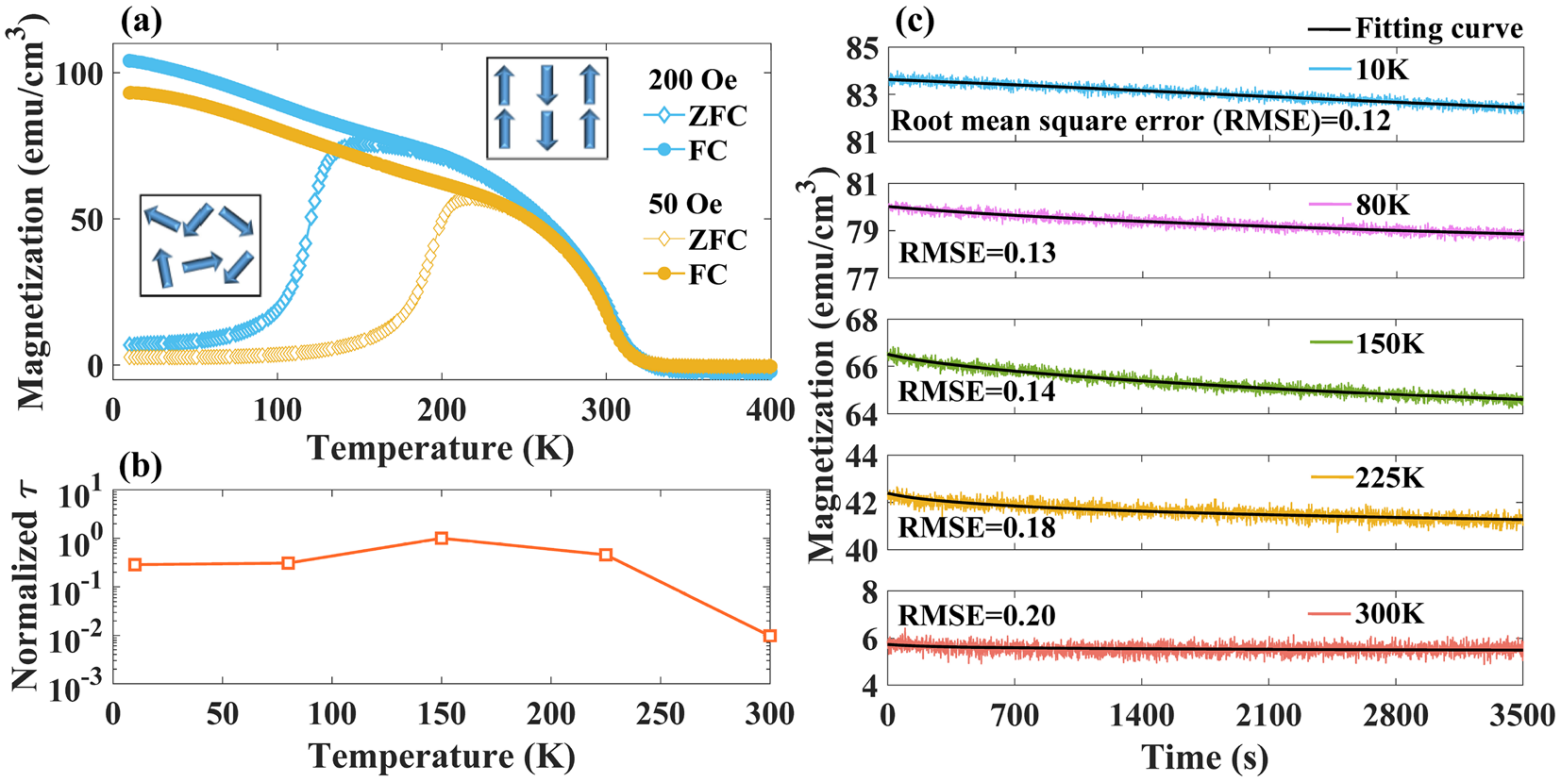
(**a**) Temperature dependence of magnetization of LFCS film measured by ZFC and FC procedures under the applied magnetic fields of 50 Oe and 200 Oe. (**b**) Normalized $\tau$ in FC procedure at 10, 80, 150, 225, and 300 K under the applied magnetic fields of 100 Oe. (**c**) The magnetic relaxation data with the fitting curve at 10, 80, 150, 225, and 300 K under the applied magnetic fields of 100 Oe.

Figure 1a shows the temperature dependence of magnetization in zero-field-cooling (ZFC) and field-cooling (FC) procedures. The irreversibility given by the divergence between ZFC and FC magnetization is a characteristic of spin glass indicating spin freezing state at temperature lower than *T*_*g*_. As illustrated at inset of Fig. 1a, randomly spin frozen state and ferrimagnetic state are realized at low temperature under ZFC and FC protocols, respectively. Prior to the measurements, the sample was heated up to 400 K for demagnetization to ensure uniformity of the initial state. Thereafter, it was cooled from 400 to 10 K in the absence of field, and the magnetization was recorded based on the ZFC procedure upon heating to 400 K in the weak fields (that is, 50 or 200 Oe). Subsequently, the magnetization was recorded upon cooling to 10 K based on the FC procedure. The magnetization in the ZFC shows a steep decrease at low temperatures that is a characteristic of spin glass. The ZFC curves exhibit cusps at blocking temperatures that are defined as the spin-freezing temperature *T*_g_. The *T*_g_ shifts systematically from 220 to 150 K as the applied magnetic field increases from 50 to 200 Oe that is a typical characteristic of spin cluster glass. Generally, the time-dependent magnetic relaxation can be fitted based on the stretched exponential function^39^:

1 $${M(t)=M}_{0}+{M}_{1}\mathrm{exp}\left[-{\left(\frac{t}{\tau [A(t)]}\right)}^{\beta [A(t)]}\right],$$

where ${M}_{0}$ and ${M}_{1}$ are constants which can reflect the glassy component of magnetization and initial remnant magnetization. $\beta$ is the stretching exponent. $\tau$ represents the relaxation time constant that reflects the duration of the relaxation behavior. $A$ is the amplitude of external magnetic field, which can influence the $\tau$ and $\beta$. Because the initialization of the curve fit affects the specific value of T, we compared the order of $\tau$ at different temperatures under the normalization condition. As shown in Fig. 1 (b), among the measured temperature conditions of LFCS, $\tau$ reaches the maximum value at 150 K with an external magnetic field of 100 Oe. With an increase in temperature over 190 K which is the *T*_g_ under applied magnetic field of 100 Oe^39^, $\tau$ gradually decreases. At 300 K, $\tau$ decreases by two orders of magnitude compared with $\tau$ at 150 K. This can be attributed to the ferrimagnetic behavior of LFCS at room temperature^39^, which reveals that magnetization is independent of time. If the temperature drops to 80 K and 10 K, $\tau$ is significantly reduced by nearly an order of magnitude, owing to the deeper frozen state of the system at lower temperatures. Figure 1 (c) displayed the experimental magnetic relaxation data with the fitting curve, which is more intuitive to reveal the effect of $\tau$ on spin glass relaxation. The index used to assess the goodness of fit is the root mean square error. The small RMSE given in Fig. 1c shows the accuracy of our fitting calculation.

### Implementation of reservoir computing

**Figure 2:**
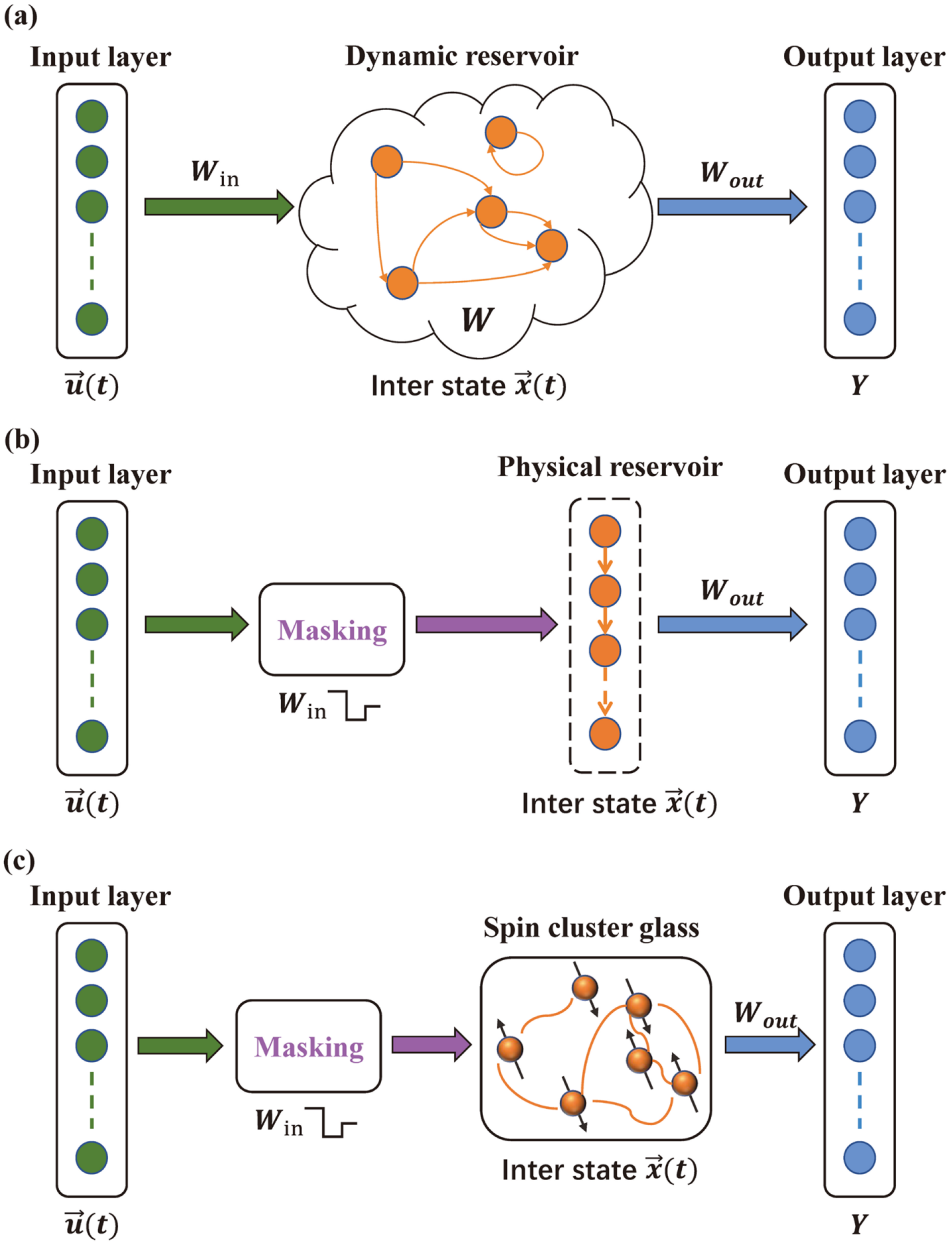
(**a**) Schematic of traditional reservoir computing. After receiving the input information, the reservoir state is dynamically updated internally. (**b**) Schematic of physical reservoir computing with time-multiplexing approach. The physical reservoir is completed by time multiplexing the physical system response. Each unit in the physical reservoir is equivalent to a sampling point. (**c**) Schematic of physical reservoir computing based on spin cluster glass. Connection of its internal nodes is determined by the complex interaction among spins of the material.

Similar to the simplest artificial neural network, traditional reservoir computing comprises three basic parts^50^: the input layer, intermediate layer (reservoir), and output layer, as illustrated in Fig. 2a. At a certain time $t$, the state of the reservoir is described by the following formula:

2 $$\overrightarrow{x}\left(t\right)=f\left[{W}_{\mathrm{in}}\overrightarrow{u}\left(t\right)+W\overrightarrow{x}\left(t-1\right)\right],$$

where $\overrightarrow{x}$ and $\overrightarrow{u}$ are the state vector and input vector of the reservoir, respectively. Suppose $J$ and $L$ are the dimensions of the input and output layers respectively, and $K$ is the number of internal nodes in reservoir. ${W}_{\mathrm{in}}$ is the $K$-by-$J$ input weight matrix. $f$ and $W$ are the nonlinear mapping function and $K$-by-$K$ internal connection matrix of the reservoir, respectively. If the matrix containing $\overrightarrow{x}\left(t\right)$ at all time steps is defined as $X$, the relationship between $X$ and the target data matrix $Y$ can be written as follows:

3 $$Y={W}_{\mathrm{out}}X,$$

where ${W}_{\mathrm{out}}$ is the $L$-by-$K$ output weight matrix and the training target. Once ${W}_{\mathrm{out}}$ is obtained, one can use Eqs. (2) and (3) to obtain the target results with any input.

In contrast to traditional reservoir computing implemented by software, the physical reservoir computing system with time-multiplexing approach depicted in Fig. 2b relies on a unique technique called masking to realize the function of the input matrix *W*_in_^51,52^. However, in the following short-term memory tasks, it is not necessary to preprocess the input by masking. Moreover, to reduce energy consumption, a physical reservoir computing system generally has only one physical unit. Therefore, it is necessary to sample the continuous response of the physical system and treat each sampling point as a virtual node^33^. It is noteworthy that $f$ and $W$ are determined by the inherent characteristics of the spin cluster glass as a physical reservoir computing system, as shown in Fig. 2c.

**Figure 3:**
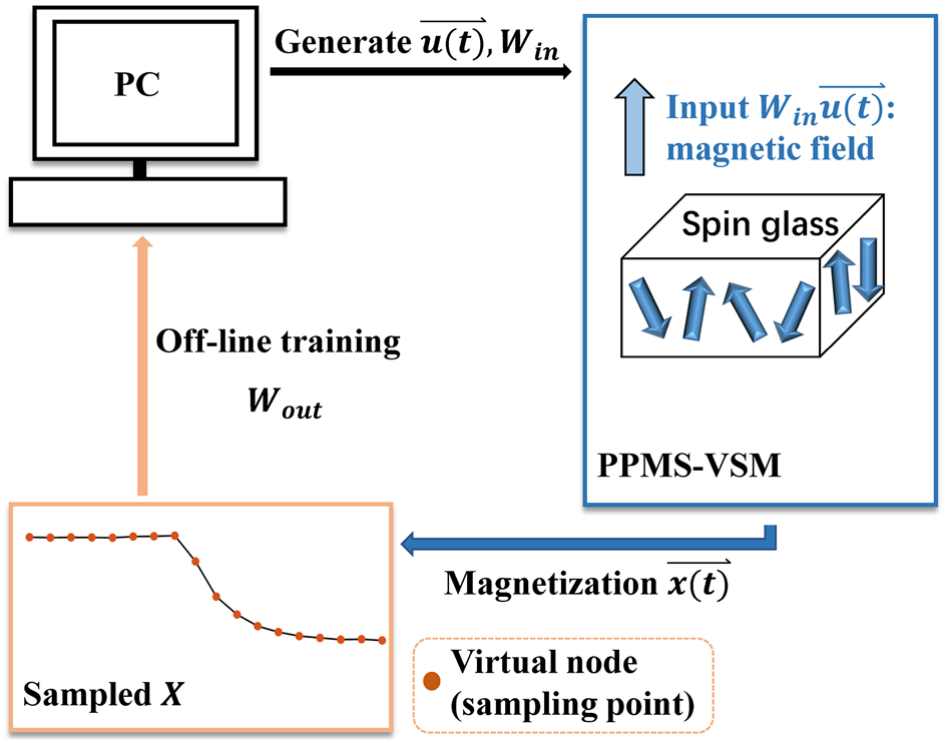
Schematic of reservoir computing experiment implementation on spin cluster glass material.

Figure 3 shows the experimental schematic of realizing the above training process in spin cluster glass material. Firstly, the input training sequence $\overrightarrow{u}\left(t\right)$ and input weight matrix ${W}_{\mathrm{in}}$ are generated by computer. Then, the masked input sequence ${W}_{\mathrm{in}}\overrightarrow{u}\left(t\right)$ is converted into a series of applied magnetic field signal and the magnetic relaxation is recorded as $\overrightarrow{x}\left(t\right)$ by the physical property measurement system with vibrating sample magnetometer (PPMS-VSM). After collecting all the $\overrightarrow{x}\left(t\right)$ containing at all time step, we can obtain $X$. Finally, according to the initial determined target data matrix $Y$ and obtained $X$, ${W}_{\mathrm{out}}$ can be calculated offline in the computer.

### Short-term memory benchmark

Short-term memory, also known as fading memory, is the premise for reservoir computing to complete the above training correctly^33^. Similar to the principle of transmitter depletion in the human brain^53,54^, short-term memory is the key that enables reservoir computing to process and encode the received information. To test the short-term memory ability, an input binary sequence ${s}_{in}\in [0, 1]$ should be randomly generated. In our work, the binary input ${s}_{in}$ follows the Bernoulli distribution that takes 0 and 1 with probabilities $p=50\%$ and $(1-p)=50\%$, respectively. Thereafter, the target sequence $y(N, d)$ can be obtained using the following formula:

4 $$y\left(N,d\right)={s}_{\mathrm{in}}\left(N-d\right),$$

where $N$ and $d$ are the $N$ th data and delay distance, respectively, that are positive integers. In this study, ${s}_{\mathrm{in}}$ contains 200 randomly generated binary codes, 30 of which are used for washout, 130 for training, and 40 for verification. In order to avoid the error caused by the large linear correlation of some variables in $X$, we use the ridge regression method to calculate *W*_out_^55^. The calculation formula is as follows:

5 $${W}_{out}={({X}^{T}X+\lambda I)}^{-1}{X}^{T}Y,$$

where $I$ represents the identity matrix and $\lambda$ is a constant which is equal to ${10}^{-8}$. Thereafter, the prediction output ${y}_{p}(N)$ corresponding to the test set can be obtained using Eq. (3). To characterize the performance of the short-term memory task, the square of the correlation coefficient between the predicted target and real target is calculated as^33^:

6 $${{r}_{\mathrm{STM}} (d)}^{2}=\frac{Cov{[{y}_{\mathrm{p}}\left(N\right), y\left(N,d\right)]}^{2}}{Cov[{y}_{\mathrm{p}}\left(N\right)]\times Cov[y\left(N,d\right)]},$$

where $Cov[{y}_{\mathrm{p}}\left(N\right), y\left(N,d\right)]$ is the covariance between ${y}_{\mathrm{p}}\left(N\right)$ and $y\left(N,d\right)$. $Cov[{y}_{\mathrm{p}}\left(N\right)]$ and $Cov[y\left(N,d\right)]$ are equivalent to $Cov[{y}_{\mathrm{p}}\left(N\right),{y}_{p}\left(N\right)]$ and $Cov[y\left(N,d\right),y\left(N,d\right)]$, respectively. ${{r}_{STM} (d)}^{2}\in [\mathrm{0,1}]$ is proportional to the short-term memory ability of reservoir computing when the delay distance is $d$. Thereafter, the short-term memory capacity can be obtained by

7 $$C=\sum_{d=1}^{{d}_{max}}{{r}_{\mathrm{STM}} (d)}^{2}.$$

In this work, when ${d}_{max}>20$, the value of ${{r}_{\mathrm{STM}} (d)}^{2}$ is less than 0.01. Hence, we take ${d}_{max}=20$. To ensure reliability, 10 independent experiments were performed. It is noteworthy that each binary input is a continuous signal of equal duration in the physical reservoir computing. Therefore, it is necessary to sample the output signal of the physical reservoir computing for the training process. In this short-term memory test, each binary code lasts for 100 s and is input by PPMS-VSM in the form of an in-plane magnetic field. In addition, the binary code {0, 1} is mapped to {0, $A$ Oe}, where $A$ is the amplitude of the magnetic field. For the duration of each code, the amplitude of the input magnetic field $A$ consists of a bias term ${A}_{bias}$ and binary random data ${A}_{p}{s}_{in}$:

8 $$A={A}_{bias}+{A}_{p}{s}_{in}.$$

The sampling frequency in the training process was 1 Hz.

## Results and discussion

### Simulation evaluation for short-term memory capability of spin glass relaxation

In order to numerically test the short-term memory ability of the relaxation term of the spin glass, we first randomly generate binary sequence with 200 codes. Each code contains 100 points to simulate the discrete sampling physical signal, which indicates that the virtual node number of the spin glass system is 100. Thereafter, we determine the time of each positive magnetization relaxation and negative magnetization relaxation according to the binary code. According to Eq. (1), we can then obtain the relaxation sequence of spin glass for the corresponding binary input sequence. Notably, as the premise of simulation, we need to carry out the magnetization relaxation measurement experiment described in Section "Spin cluster glass of Lu3Fe4Co0.5Si0.5O12" to determine the relaxation parameters under specific temperature scheme. Since ${M}_{0}$ and ${M}_{1}$ can be normalized during training, accurate $\tau$ and $\beta$ are more important. Because the magnetization relaxation characteristic of spin glass is the strongest near *T*_g_, we extracted $\tau$ and $\beta$ at a constant temperature of *T*_g_ using Eq. (1). The obtained $[\tau ,\beta ]$ values for the FC and ZFC relaxations under an external applied magnetic field of $A=100$ Oe are [2741 s, 0.60] and [3848 s, 0.53], respectively^39^. In contrast, the short-term memory benchmark was also tested on tanh function. The tanh function is representative of the threshold activation function in reservoir computing^56^ as well as other artificial neural networks^57–59^, which is used to simulate the excitation and inhibition of human neurons. In this work, tanh can be used to numerically simulate the magnetodynamics of LuIG.

**Figure 4:**
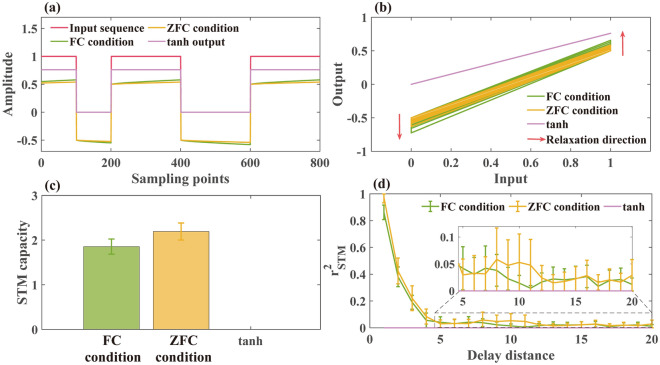
(**a**) Input binary sequence and the output from the spin glass relaxation term of FC condition, ZFC condition, and tanh function, respectively. Each binary code is sampled into 100 discrete points. (**b**) Relationship curve between input binary sequence and the output from each system. (**c**) short-term memory capacity and (**d**) ${{r}_{\mathrm{STM}} (d)}^{2}$ of each system.

The time-domain responses of three systems to random binary sequences are shown in Fig. 4a. It can be seen that when the input signal is switched between 0 and 1, the output of the tanh function reaches the steady state instantaneously without any relaxation transition. Different from the tanh without relaxation behavior, spin glass in the form of ZFC and FC form shows a relatively long-time relaxation duration. Generally, the shorter the relaxation time, the weaker is the short-term memory capability^29^. Moreover, in Fig. 4b, the relationship between the input and output of the spin glass forms a loop, whereas that of the tanh function forms a straight line. The loop relationship between input and output is the embodiment of the system memory effect^60–62^. Therefore, from Fig. 4a and b, we can qualitatively understand the short-term memory capability comparison of the three systems. Specifically, the tanh function has no short-term memory capability. It should be noted that when the relaxation time $\tau$ is shortened, the relaxation of spin glass will weaken and approach the output by tanh function. Thereby, one can expect that the short-term memory capacity of spin glass will decrease when the temperature is significantly higher / lower than *T*_g_.

For further quantitative analysis, Fig. 4c and d show the short-term memory benchmark test results of the three systems. Consistent with the qualitative analysis, the short-term memory capability of spin glass in both the ZFC and FC relaxation states is better than that of the tanh function, which is 0. In Fig. 4d, ${{r}_{STM} (d)}^{2}$ of the spin glass under both ZFC and FC condition first decreases with the delay distance, and thereafter fluctuates near 0. Although there is no significant difference in the attenuation rate of ${{r}_{STM} (d)}^{2}$, the ${{r}_{STM} (d)}^{2}$ of ZFC state is higher than that of FC state at $d=1$, which contributes to the short-term memory difference between the two conditions. In essence, the different short-term memory capacities under ZFC and FC condition can be attributed to different magnetization relaxation processes caused by different external magnetic field conditions. Specifically, under the condition of FC, the magnetization of the spin glass is subject to additional constraints compared with that of ZFC, which also weakens the response to the binary input, resulting in a lower short-term memory capacity. From the simultaneous relaxation performance, the relaxation amplitude of spin glass in FC is less than that in ZFC^63^.

### Experimental testing for short-term memory capability of spin glass relaxation

**Figure 5:**
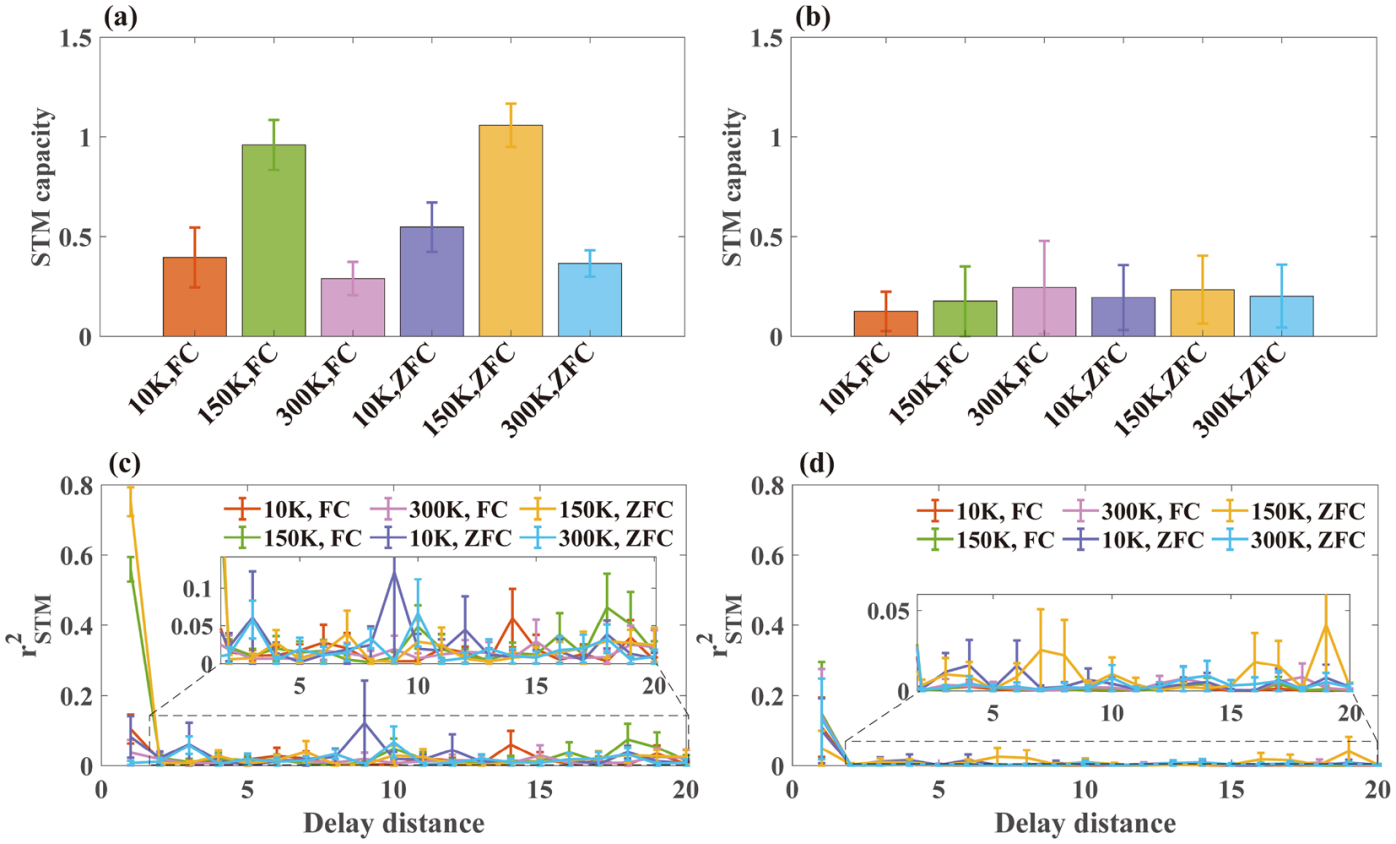
Short-term memory capacities of (**a**) LFCS and (**b**) LuIG under different temperature and cooling schemes. ${{r}_{\mathrm{STM}} (d)}^{2}$ of (**c**) LFCS and (d) LuIG under different temperature and cooling schemes. The input binary sequence is mapped from {0, 1} to {0, 100 Oe}.

When $A=100$ Oe, the short-term memory benchmark was experimentally tested on LFCS and LuIG. Figure 5a and b show the short-term memory capacities of the two materials at different temperatures and cooling conditions. First, it can be seen that for the LFCS of ZFC or FC relaxation, its short-term memory capacity is the best at 150 K. This can be explained by the effect of the change in thermal energy on the height of the potential barrier resulting from the multivalley potential^34^. At a low temperature of 10 K, the potential barrier is high, and the spin state is completely frozen, with almost no change in the magnetic field. In contrast, at temperatures around 300 K, the potential barrier reduces, the metastable state is lost, and the spin-glass-like characteristic is lost. Therefore, the short-term memory capacity is expected to be small at 10 K and 300 K. However, at around 150 K, the temperature is slightly less than the spin-freezing temperature in the magnetic field^64^, therefore the short-term memory capacity is expected to be larger because the magnetic field causes a slow magnetic relaxation that slowly exceeds the potential barrier of the magnetic state. Similar to the simulation results in Fig. 5 (a), the short-term memory capacity of FC relaxation at 150 K is lower than that of ZFC. Moreover, from Fig. 5 (c), one can expect the difference in short-term memory capacity under the two conditions is mainly contributed by ${{r}_{\mathrm{STM}} (d=1)}^{2}$. Therefore, the short-term memory capability of LFCS under ZFC relaxation is better than that of FC relaxation that is consistent with the simulation results. Notably, a delay distance of 1 means a time scale of 1 s, which is the minimum measurement interval of PPMS-VSM. For the traditional spintronics RC, its short-term memory can only maintain microsecond level^29^. In contrast, the LFCS can maintain the short-term memory ability at the second level. It implies that even for the high-speed signal in a shorter time scale, the spin cluster glass can still be expected to provide considerable short-term memory capacity.

Furthermore, for LuIG, the short-term memory capability should be close to the tanh function in theory because of the extremely short relaxation time of ferrimagnetic materials^65^. In Fig. 5b, one can show that the short-term memory capacity fluctuates sharply under the tested conditions. In Fig. 5d, for almost every $d$, the error of ${{r}_{\mathrm{STM}} (d)}^{2}$ is large compared to its average value. Hence, it can be considered that the short-term memory capacity of LuIG is extremely low and the measurement result indicates the error fluctuation caused by the environment and equipment disturbance.

**Figure 6:**
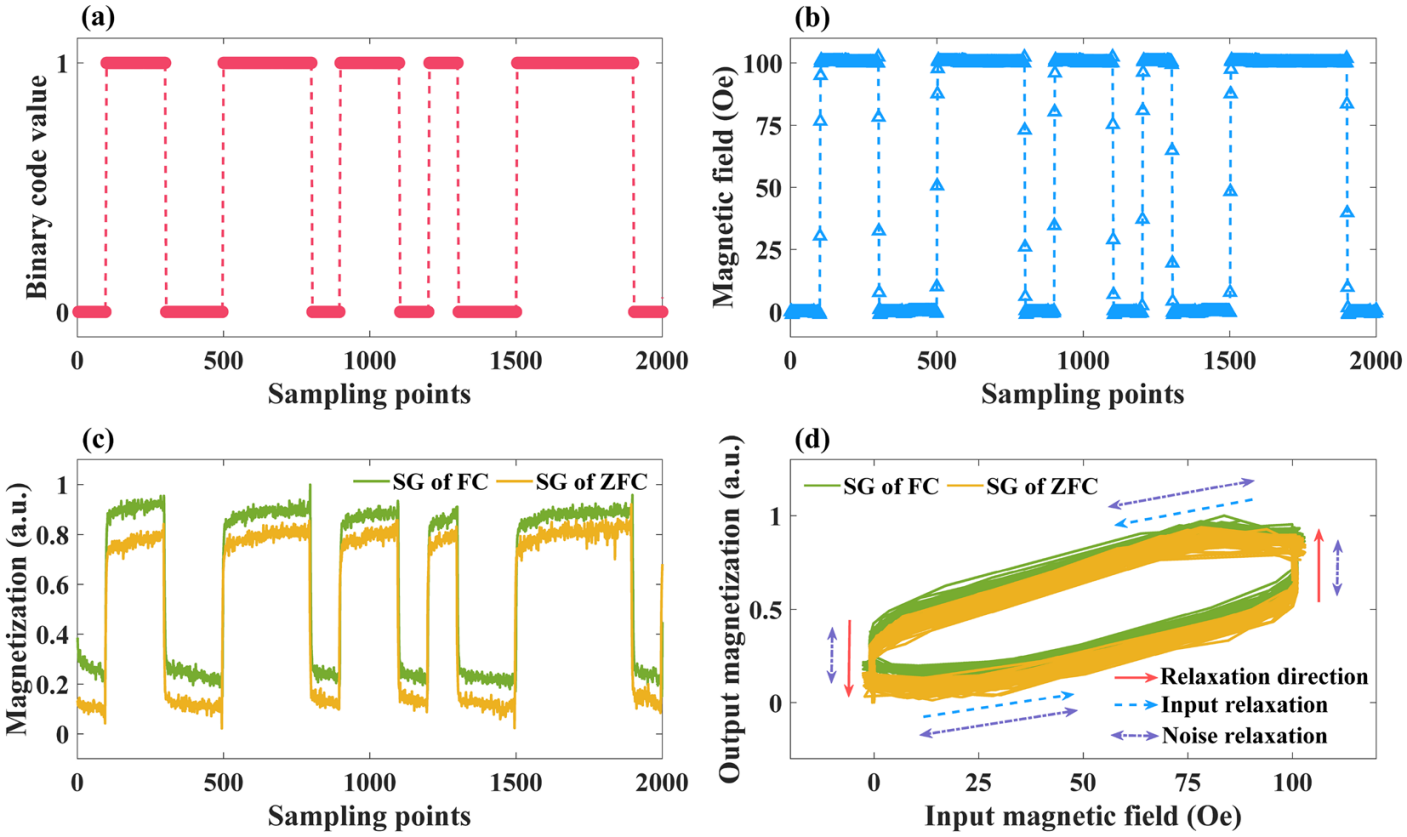
(**a**) Randomly generated binary sequence. In the ideal case, binary code only takes two values—0 and 1. (**b**) Input magnetic field applied by PPMS-VSM according to the binary sequence. The scattered points between 0 and 100 Oe are caused by the non-transient magnetic field applied by the system. (**c**) Magnetization (a.u.) of FC and ZFC relaxation in LFCS at 150 K. (**d**) Relationship curve between input magnetic field and the output magnetization (a.u.) of FC and ZFC relaxation in LFCS.

Although the aforementioned results preliminarily prove that LFCS has a short-term memory capacity around 2 at 150 K, there is a noticeable gap between the experimental results and simulation. To further analyze the source of this gap, the ideal input and the input magnetic field applied by PPMS-VSM under the same binary test sequence are depicted in Fig. 6a and b. In the ideal case, the binary sequence should be instantaneously switched between 0 and 1, without transition values. In the actual case, the externally applied physical signal cannot be changed instantaneously, which will produce a transition state, such as the scattered points in Fig. 6b. The positions of these scattered points are irregular, and the intervals are not uniform; thus, random relaxation independent of input information is introduced. In addition, it can be observed that in the magnetization curves measured by PPMS-VSM (Fig. 6c), regardless of FC or ZFC relaxation, the magnetization of spin glass is markedly disturbed by noise during the relaxation process. This implies that noise also introduces random relaxation independent of the input information. The directions of input relaxation and noise relaxation are shown in Fig. 6d. In the ideal condition of the simulation, the input–output curve with short-term memory capability should form a rectangle (Fig. 4b). However, the random relaxation caused by the input and noise obviously distorted the input–output relationship curve. Comparing the durations of the two types of random relaxation, when $A=100$ Oe, one can expect that the duration of the input relaxation is approximately 3–4 s, which is significantly shorter than the duration (100 s) of a binary code. However, noise relaxation is always present. In addition, in a previous study^28^, the short-term memory capacity of physical reservoir computing was increased by approximately 1 after the system noise was suppressed. Therefore, it can be perceived that the main reason behind the difference in the short-term memory capability of spin glass between the experimental and simulation results, is the random relaxation introduced by noise. For the input relaxation, it is difficult to completely eliminate its negative influence due to the inevitable transition process of the physical system. As a feasible method, we can reduce the effect of input relaxation by increasing the duration of each input code to decrease the proportion of the transition process in the whole signal input process. For reducing the impact of noise, filters and time-domain averaging techniques are effective methods which has been widely used in other reservoir computing systems ^28,66^.

**Figure 7:**
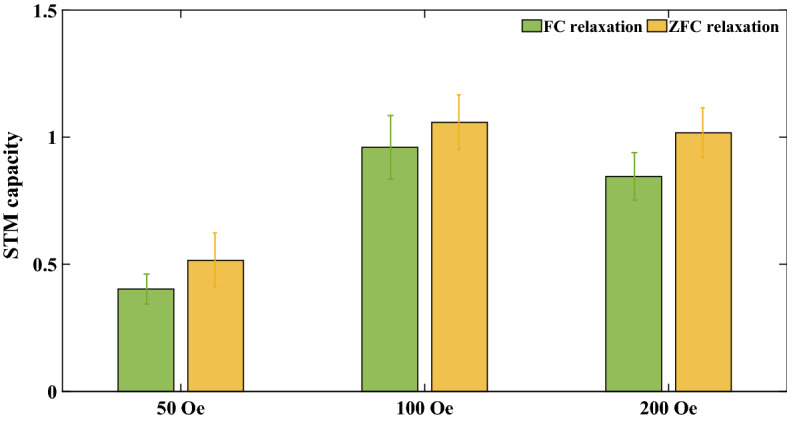
Short-term memory capacity of LFCS when the input binary sequence is mapped from {0, 1} to (**a**) {0, 50 Oe}, (**b**) {0, 100 Oe}, and (**c**) {0, 200 Oe}.

Finally, we determine whether the mapping relationship between the binary code and the external magnetic field, that is, the value of $A$, affects the short-term memory capability of the spin glass. We experimentally evaluated the performance of spin glass on the short-term memory benchmark at 150 K when {0, 1} binary codes were mapped to {0, 50 Oe}, {0, 100 Oe} and {0, 200 Oe}, respectively. The test results are shown in Fig. 7.

Figure 7 shows that with an increase in the external magnetic field, the short-term memory capacity of spin glass first increases, subsequently reaching saturation or even slightly decreasing. When the magnetic field amplitude is small, spin glass is more susceptible to noise due to weaker magnetization. However, with the increase in the magnetic field, the transition time in the input signal is extended, that is, the input relaxation intensifies. The stronger input relaxation limits the growth of short-term memory capacity, which results in the trend shown in Fig. 7. Notably, although the short-term memory capacity of LFCS is saturated to about 1 in a noisy environment, this value should not be considered a small value. For many traditional spintronics reservoir computing device, the short-term memory capacity in noisy environment is about 2 to 3^28,29^. Because the overall short-term memory capacity and nonlinearity of a physical reservoir computer are jointly contributed by each component ^28,29,67^, using spin glass as the basic material has the potential to increase the overall short-term memory capacity of traditional spintronics reservoir computing devices by about 1/3 to 1/2 at most.

## Conclusion

Based on the short-term memory benchmark commonly used in physical reservoir computing, we quantitatively evaluated the short-term memory ability of magnetization in LFCS, both numerically and experimentally. Under ideal conditions, the short-term memory capability of LFCS is approximately 2 and comparable to some physical reservoir computing systems with a single node^28,33^. Although in the experiment, we found that the additional irregular relaxation caused by noise and the measurement system degenerate the short-term memory capacity of LFCS, its short-term memory capacity is still larger than that of ferrimagnetic LuIG without spin glass behavior.

It is worth noting that the above conclusions are not intended to encourage the development of a physical reservoir computing system that depends only on the relaxation behavior of spin cluster glass materials. Rather, the conclusion of this study should be understood as providing an improved basic material candidate for spintronic reservoir computing based on magnetodynamic relaxation. When combining spin glass with existing spintronic reservoir computing systems, some additional physical considerations need to be taken into account according to the specific system. For example, if combing the spin glass with spin wave equipment, it should be careful to consider the change of damping constant after replacing materials, which affects the shape designing of the spin cluster glass. Because the nonlinearity and short-term memory capability of the spintronic reservoir computing result from the interaction of various components in the system^28,29^, choosing a material that can provide short-term memory capability will undoubtedly be more conducive to the enhancement of spintronic reservoir computing performance. However, an optimal short-term memory capacity of LFCS is obtained near 150 K. Therefore, it is a challenging and an important task in future research to realize materials exhibiting spin glass behavior at higher temperatures.

## Data Availability

The datasets measured during and/or analyzed during the current study are available from the corresponding author upon reasonable request.
